# A 23-year retrospective investigation of *Salmonella* Typhi and *Salmonella* Paratyphi isolated in a tertiary Kathmandu hospital

**DOI:** 10.1371/journal.pntd.0006051

**Published:** 2017-11-27

**Authors:** Raphaël M. Zellweger, Buddha Basnyat, Poojan Shrestha, Krishna G. Prajapati, Sabina Dongol, Paban K. Sharma, Samir Koirala, Thomas C. Darton, Christiane Dolecek, Corinne N. Thompson, Guy E. Thwaites, Stephen G. Baker, Abhilasha Karkey

**Affiliations:** 1 The Hospital for Tropical Diseases, Wellcome Trust Major Overseas Programme, Oxford University Clinical Research Unit, Ho Chi Minh City, Vietnam; 2 Oxford University Clinical Research Unit, Patan Academy of Health Sciences, Kathmandu, Nepal; 3 Centre for Tropical Medicine and Global Health, Oxford University, Oxford, United Kingdom; 4 Global Antibiotic Resistance Partnership, Centre for Disease Dynamics Economics and Policy, Washington DC, Unites States of America; 5 Patan Academy of Health Sciences, Patan Hospital, Kathmandu, Nepal; 6 Department of Infection, Immunity and Cardiovascular Disease, University of Sheffield, Sheffield, United Kingdom; 7 The Department of Medicine, University of Cambridge, Cambridge, United Kingdom; Massachusetts General Hospital, UNITED STATES

## Abstract

**Background:**

*Salmonella* serovars Typhi (*S*. Typhi) and Paratyphi A (*S*. Paratyphi A), the causative agents of enteric fever, have been routinely isolated organisms from the blood of febrile patients in the Kathmandu Valley since the early 1990s. Susceptibility against commonly used antimicrobials for treating enteric fever has gradually changed throughout South Asia since this time, posing serious treatment challenges. Here, we aimed to longitudinally describe trends in the isolation of *Salmonella enterica* and assess changes in their antimicrobial susceptibility in Kathmandu over a 23-year period.

**Methods:**

We conducted a retrospective analysis of standardised microbiological data from April 1992 to December 2014 at a single healthcare facility in Kathmandu, examining time trends of *Salmonella*-associated bacteraemia and the corresponding antimicrobial susceptibility profiles of the isolated organisms.

**Results:**

Over 23 years there were 30,353 positive blood cultures. *Salmonella enterica* accounted for 65.4% (19,857/30,353) of all the bacteria positive blood cultures. *S*. Typhi and *S*. Paratyphi A were the dominant serovars, constituting 68.5% (13,592/19,857) and 30.5% (6,057/19,857) of all isolated *Salmonellae*. We observed (i) a peak in the number of Salmonella-positive cultures in 2002, a year of heavy rainfall and flooding in the Kathmandu Valley, followed by a decline toward pre-flood baseline by 2014, (ii) an increase in the proportion of *S*. Paratyphi in all *Salmonella*-positive cultures between 1992 and 2014, (iii) a decrease in the prevalence of MDR for both *S*. Typhi and *S*. Paratyphi, and (iv) a recent increase in fluoroquinolone non-susceptibility in both *S*. Typhi and *S*. Paratyphi isolates.

**Conclusions:**

Our work describes significant changes in the epidemiology of *Salmonella enterica* in the Kathmandu Valley during the last quarter of a century. We highlight the need to examine current treatment protocols for enteric fever and suggest a change from fluoroquinolone monotherapy to combination therapies of macrolides or cephalosporins along with older first-line antimicrobials that have regained their efficacy.

## Author summary

Aiming to understand the epidemiology and changing patterns of the *Salmonella enterica* serovars Typhi and Paratyphi A within a single healthcare facility in Kathmandu, we retrospectively analysed 23 years of microbiological blood culture data. From 224,741 blood cultures performed, 30,353 were confirmed to be positive for pathogenic bacteria, of which *Salmonella enterica* accounted for 65.4% (19,857/30,353) of all the bacteria positive blood cultures. *S*. Typhi and *S*. Paratyphi A were the dominant serovars, constituting 68.5% (13,592/19,857) and 30.5% (6,057/19,857) of all isolated *Salmonellae*. We observed that *S*. Typhi and *S*. Paratyphi A remained the leading cause of bacterial febrile illness since the 1990s. Additionally, antimicrobial resistance is a major public health challenge, and resistance against the antimicrobials most commonly used for treating enteric fever has developed over the last two decades. In this analysis we were able to document a decrease in the number of *Salmonella*-positive cultures from 2002 to 2014 and an increase in the proportion of *S*. Paratyphi A in all *Salmonella*-positive cultures between 1992 and 2014. Concurrently, we also observed a decrease in MDR for *S*. Typhi and *S*. Paratyphi and a recent increase in fluoroquinolone non-susceptibility in both serovars.

## Introduction

Enteric (typhoid) fever is a systemic disease caused by *Salmonella* serovars Typhi (*S*. Typhi) and Paratyphi A (*S*. Paratyphi A). Global estimates suggest that 12 to 27 million new cases of enteric fever occur annually resulting in 130,000 to 220,000 deaths, which predominantly occur in low-middle income countries (LMICs) [1–3]. Enteric fever has been a public health concern in Nepal for some time, with *S*. Typhi and *S*. Paratyphi A consistently being regularly isolated from the blood of febrile patients in the Kathmandu Valley since the early 1990s [4–6]. The sustained high prevalence of *S*. Typhi and *S*. Paratyphi A in the Kathmandu Valley has been attributed to a contaminated water supply and poor sanitation [6].

Enteric fever is an infection that requires antimicrobial therapy, but antimicrobial resistance (AMR) hinders effective treatment, prolonging the duration of fever and leaving patients at risk of further complications. As a consequence of horizontal acquisition of resistance genes and point mutations, the antimicrobial susceptibility profiles of *S*. Typhi and *S*. Paratyphi A have changed substantially in Asia over the past 30 years. Organisms resistant to ampicillin, chloramphenicol, and co-trimoxazole (multidrug-resistant, MDR) first appeared in the 1980s and 1990s and were associated with large focal outbreaks [7]. The emergence and persistence of resistance against these antimicrobials diverted clinical practice towards more frequent use of fluoroquinolones to treat enteric fever [8]. This shift towards the use of fluoroquinolones was predictably followed by a general decline in susceptibility against these antimicrobials through sequential mutations in the genes encoding the enzymes targeted by fluoroquinolones [8].

Understanding longitudinal changes in isolation patterns of *Salmonella* serovars and fluctuations in their corresponding antimicrobial susceptibility profiles in locations where enteric fever is endemic is important for disease surveillance. These data are critical for improving clinical care, updating treatment guidelines, and guiding public health interventions. Aiming to delineate trends in the isolation of invasive *Salmonella* and their corresponding antimicrobial susceptibility profiles, we conducted a retrospective analysis of 23 years of *Salmonella* isolated from blood cultures taken from patients attending a major healthcare facility in the Kathmandu Valley in Nepal.

## Material and methods

### Ethics statement

Data for this study consisted of anonymised laboratory results devoid of individual patient information or identifiers. This study was therefore part of the routine surveillance measures within this healthcare facility and ethical approval and individual informed consent were not necessary. However, a written permission, for access and analysis of the data, was sought and obtained from the Patan Hospital management.

### Study design and data collection

This study was a retrospective analysis of all blood cultures performed between April 1992 and December 2014 at Patan Hospital. Patan Hospital is located in Lalitpur Sub-metropolitan City (LSMC) within the Kathmandu Valley in central Nepal. It is one of three general hospitals in the greater metropolitan area of Kathmandu. The hospital had a capacity of 138 beds in 1992, 350 beds in 2014, a current capacity of 592 beds, and provides emergency and elective services to outpatients (approximately 200,000 outpatient visits per year) and inpatients, 90% of which live in LSMC.

For each patient, the date of blood draw, the ward in which the blood sample was taken, the result of the culture (whether positive or negative) and the susceptibility of the isolate to commonly used antimicrobials were recorded by a member of the hospital laboratory personnel. For the purposes of this investigation, all handwritten data recorded between the specified dates were transcribed into bespoke software designed by Bonfire Technologies, Nepal. All data were double entered; 10% of all entries were subjected to random inspection by a team of dedicated data entry personnel from the Nepal Family Development Foundation (NFDF). These were the source data for this study.

### Microbiology procedures and statistical analysis

Conventional manual blood cultures were performed for the majority of the data used in these analyses with the exception of the paediatric population (<14 years of age). Conventional blood cultures were performed by inoculating 3–5 ml of blood from paediatric patients and 5–8 ml of blood from adult patients into 30–50 ml of media containing tryptone soya broth containing 0.05% sodium polyanetholesulfonate. BACTEC Peds plus bottles (Becton Dickinson, Sparks, MD, USA) were used for the paediatric samples after an automated system was introduced. Conventional bottles were incubated at 37°C in a standard microbiological incubator and examined daily for growth for a period of seven days. An automated BACTEC (Becton Dickinson, MD, USA) culture system was used for paediatric samples from 2005 onwards. Organisms isolated from blood were identified using Gram staining, standard biochemical tests, and specific antisera.

Antimicrobial susceptibilities were tested at the time of isolation by the modified Kirby-Bauer disc diffusion method. Zone size interpretations were initially recorded according to the guidelines provided in the antimicrobial packaging; from 2003 zone sizes were interpreted according to the annual CLSI guidelines. Dependant on the period of isolation and the organism a range of antimicrobials were tested, including: Amikacin (AMK), amoxicillin (AMX), ampicillin (AMP), cefixime (CFM), cefotaxime (CTX), ceftriaxone (CRO), cephalexin (LEX), chloramphenicol (CHL), ciprofloxacin (CIP), trimethoprim-sulphamethoxazole (SXT), gatifloxacin (GAT), gentamicin (GEN), nalidixic acid (NAL), norfloxacin (NOR), ofloxacin (OFX), tetracycline (TET), tigecycline (TGC), tobramycin (TOB). Following antimicrobials were occasionally tested for: ampicillin-sulbactam (SAM), cefepime-tazobactam (FEP.TZB), cefoperazone (CFP), cefoperazone-sulbactam (CFP.SUL), cefoxitin (FOX), ceftriaxone-sulbactam (CRO.SUL), cloxacillin (CLO), colistin (CST), erythromycin (ERY), imipenem (IPM), meropenem (MEM), nitrofurantoin (NIT), oxacillin (OXA), penicillin (PEN), piperacillin (PIP), piperacillin-tazobactam (TZP), and teicoplanin (TEC). Resistant and intermediate isolates were grouped as “non-susceptible” and MDR was defined as non-susceptibility to ampicillin/amoxicillin, chloramphenicol, and co-trimoxazole.

All statistical analysis was performed using statistical software R, version 3.3.2[9]. Increasing or decreasing trends over time were identified using the Cox and Stuart test for trends (R-package “randtrends”). Davies’ test (R-package “segmented”) was used to detect a non-constant regression parameter (i.e. change in the slope) in linear regressions.

## Results

### General characteristics

From April 1992 to December 2014, a total of 224,741 individual patient blood samples were drawn for microbiological culture at this healthcare facility. Of these, 173,892 (77.4%) were culture-negative, 10,496 (4.7%) were positive for non-*Salmonella* bacteria, and 20,496 (9.1%) were contaminated or contained fungi (Table 1). The remaining 8.8% of samples were positive for *Salmonella enterica* sub-species I, accounting for 65.4% (19,857/30,353) of all the bacteria positive blood cultures. *S*. Typhi and *S*. Paratyphi A were the dominant serovars, constituting 68.5% (13,592/19,857) and 30.5% (6,057/19,857) of all isolated *Salmonellae*, respectively (Table 2). *S*. Paratyphi A represented 99.44% of all *S*. Paratyphi (6,057/6,091). The annual distributions of *Salmonella*-positive, positive for non-*Salmonella* bacteria, and culture-negative/contaminated blood cultures are shown in S1 Fig.

**Table 1:** Summary of blood culture results between April 1992 and December 2014.

Blood cultures (n = 224,741)	Number	Percentage of cultures
Culture negative	173,892	77.37
Contamination*/Fungus	20,496	9.12
*Salmonella enterica*	19,857	8.84
Culture positive for other bacteria	10,496	4.67

* Contaminants included Bacillus spp., Coagulase negative staphylococci (excluding those from neonates), Micrococcus spp., Acinetobacter spp. and Diptheroids.

**Table 2:** Summary of *Salmonella* positive blood cultures between April 1992 and December 2014.

Positive cultures (n = 30,353)	Number	Percentage of *S*. *enterica*	Percentage of bacteria positive
*Salmonella* Typhi	13,592	68.45	44.78
*Salmonella* Paratyphi A	6,057	30.50	19.96
*Salmonella* Paratyphi B	27	0.14	0.09
*Salmonella* Paratyphi C	3	0.02	0.01
*Salmonella* Paratyphi spp.	4	0.02	0.01
*Salmonella* Typhimurium	4	0.02	0.01
*Salmonella* Enteritidis	4	0.02	0.01
Other *Salmonella* serovars	166	0.84	0.55
**Total**	**19,857**	**100**	**65.42**

### Time trends in the isolation of *Salmonella* Typhi and *Salmonella* Paratyphi

**Fig 1:**
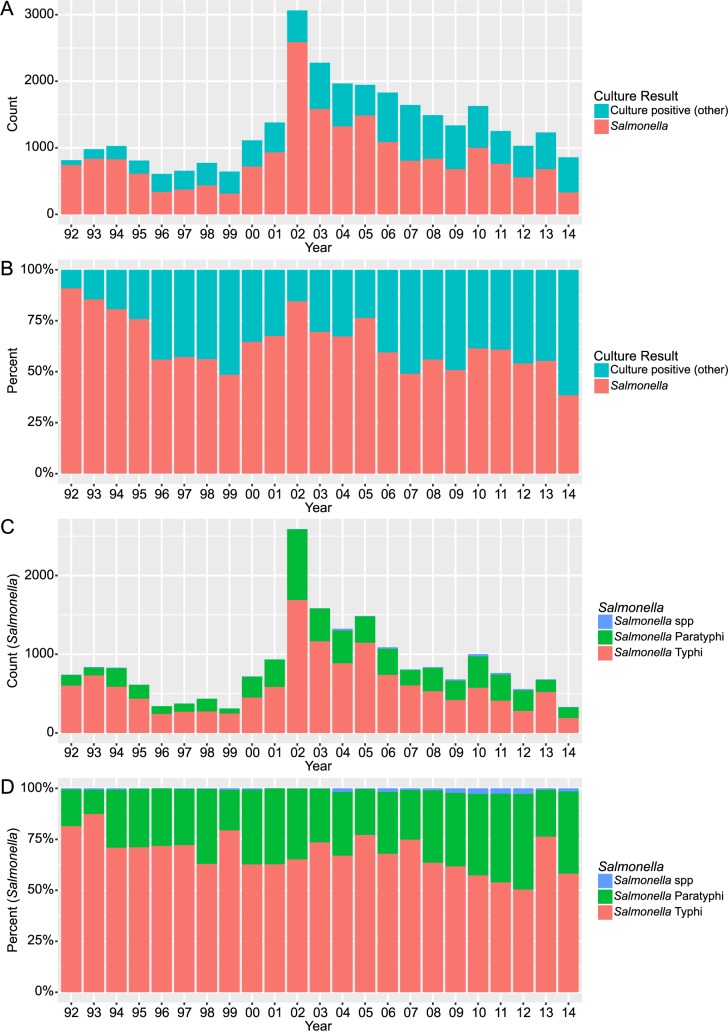
The annual distribution of bacteria positive blood cultures from 1992 to 2014. Bar graph showing the annual counts (A) and percentages (B) of *Salmonella*-positive cultures in all positive blood samples collected from 1992 to 2014. The annual counts and percentages of *S*. Typhi, *S*. Paratyphi, and *Salmonella* spp. in all *Salmonella-*positive cultures collected from 1992 to 2014 are shown in (C) and (D), respectively.

The annual distributions of *Salmonella*-positive blood cultures (in all bacteriologically positive blood cultures) are shown in Fig 1. The proportion of *Salmonella* positive blood cultures did not exhibit a uniformly increasing or decreasing trend throughout the study period. However, we observed a peak of 2,590 positive cultures in 2002, a year of unusually high rainfall and flooding in the Kathmandu Valley. Subsequently, the total number of *Salmonella*-positive cultures decreased towards pre-2002 baseline numbers and the proportion of *Salmonella*-positive blood cultures (in all bacteria-positive cultures) exhibited a significant decreasing trend from 2002 to 2014 (*p* = 0.016). During this 13-year period (2002–2014), the proportion of *Salmonella* in all bacteria positive blood cultures decreased by a mean of 2.4% (95% CI: 1.1–3.7) per year (S2 Fig). Additionally, there was a significant decreasing trend in the absolute number of blood cultures from which *Salmonella* were isolated during this corresponding period (*p* = 0.016).

*S*. Typhi was markedly the more prevalent of the two *Salmonella* serovars over the period of investigation (Fig 1). However, the proportion of *S*. Typhi (with respect to all isolated *Salmonella*) exhibited a significant decreasing trend from 1992 to 2014 (*p* = 0.033), decreasing on average by 0.81% a year (95% CI: 0.3–1.3). Conversely, the proportion of *S*. Paratyphi A (with respect to all isolated *Salmonella*) exhibited a significant increasing trend over the same time period (*p* = 0.033).

**Fig 2:**
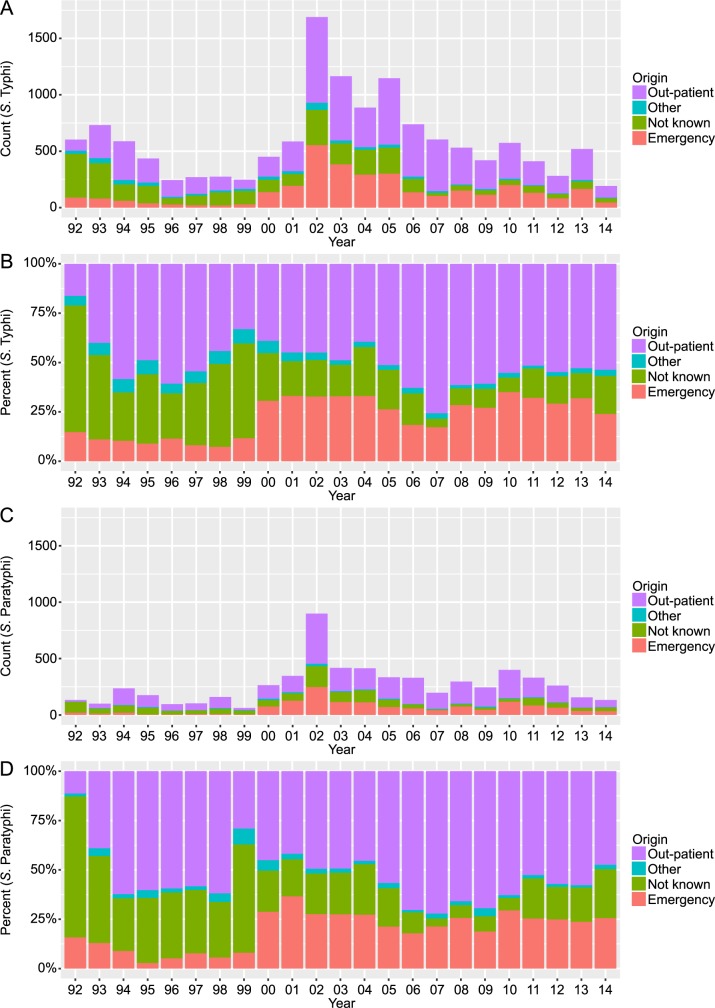
The annual distribution of ward from which *Salmonella* positive blood cultures were taken from 1992 to 2014. Annual counts (A) and percentages (B) of the ward of origin for *S*. Typhi positive cultures collected from 1992 to 2014. The annual counts and percentages of the ward of origin for the *S*. Paratyphi positive cultures collected from 1992 to 2014 are shown in (C) and (D), respectively.

To better appreciate the population presenting at this healthcare facility with enteric fever, we investigated from which department within the hospital the blood samples that were positive for *Salmonella* had originated (Fig 2). Almost half of the *S*. Typhi and *S*. Paratyphi positive cultures (6,713/13,592; 49.4% and 3,298/6,091; 54.1%, respectively) originated from the outpatient department. Notably, the proportion of *S*. Typhi and *S*. Paratyphi positive blood cultures arising from the emergency department increased from the year 2000 onwards; the emergency department continued to contribute a large proportion of *Salmonella*-positive blood samples after this year.

**Fig 3:**
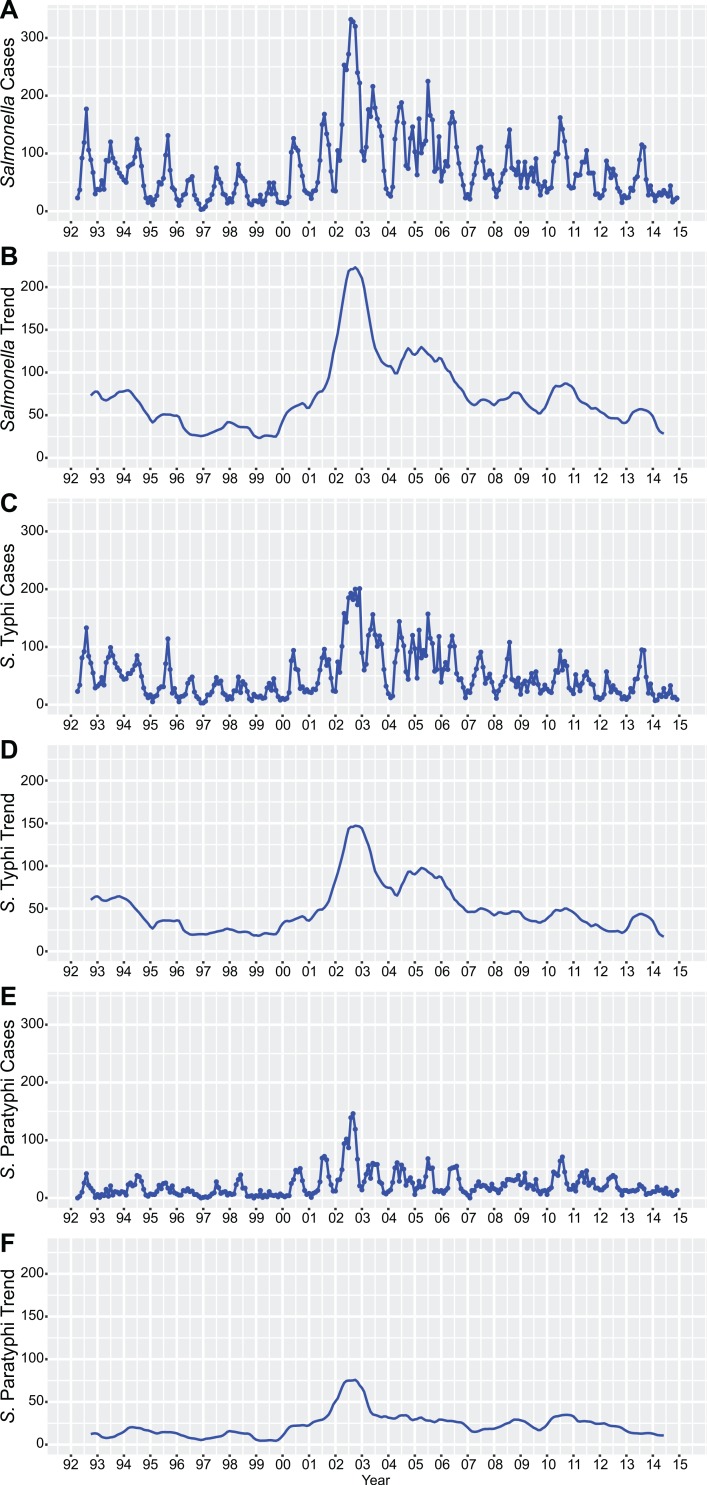
Time series for *Salmonella*-positive cultures from 1992 to 2014. Plots showing the monthly time series of all *Salmonella* positive blood cultures (absolute numbers in (A) and trends in (B)) between April 1992 and December 2014. Time series (absolute numbers and trends) for *S*. Typhi are shown in (C) and (D), and for *S*. Paratyphi in (E) and (F).

Fig 3 shows the monthly time series for the *Salmonella*-positive cultures. June, July, August, and September were the months when the majority of *Salmonella* were isolated from blood cultures. The number of *Salmonella*-positive blood cultures (all *S*. *enterica*, *S*. Typhi, and *S*. Paratyphi A) exhibited maximal autocorrelation 12 months apart, therefore confirming seasonality of *Salmonella* infections (S3 Fig). The time trends, which represent longer-term fluctuations in the number of positive blood cultures (after filtering for seasonality and random variation), exhibited major peaks for *S*. Typhi and *S*. Paratyphi in 2002, as well as smaller confined peaks for *S*. Typhi in 2005, 2010, and 2013 and *S*. Paratyphi in 2010.

### Antimicrobial susceptibility patterns of *Salmonella* Typhi and *Salmonella* Paratyphi

**Fig 4:**
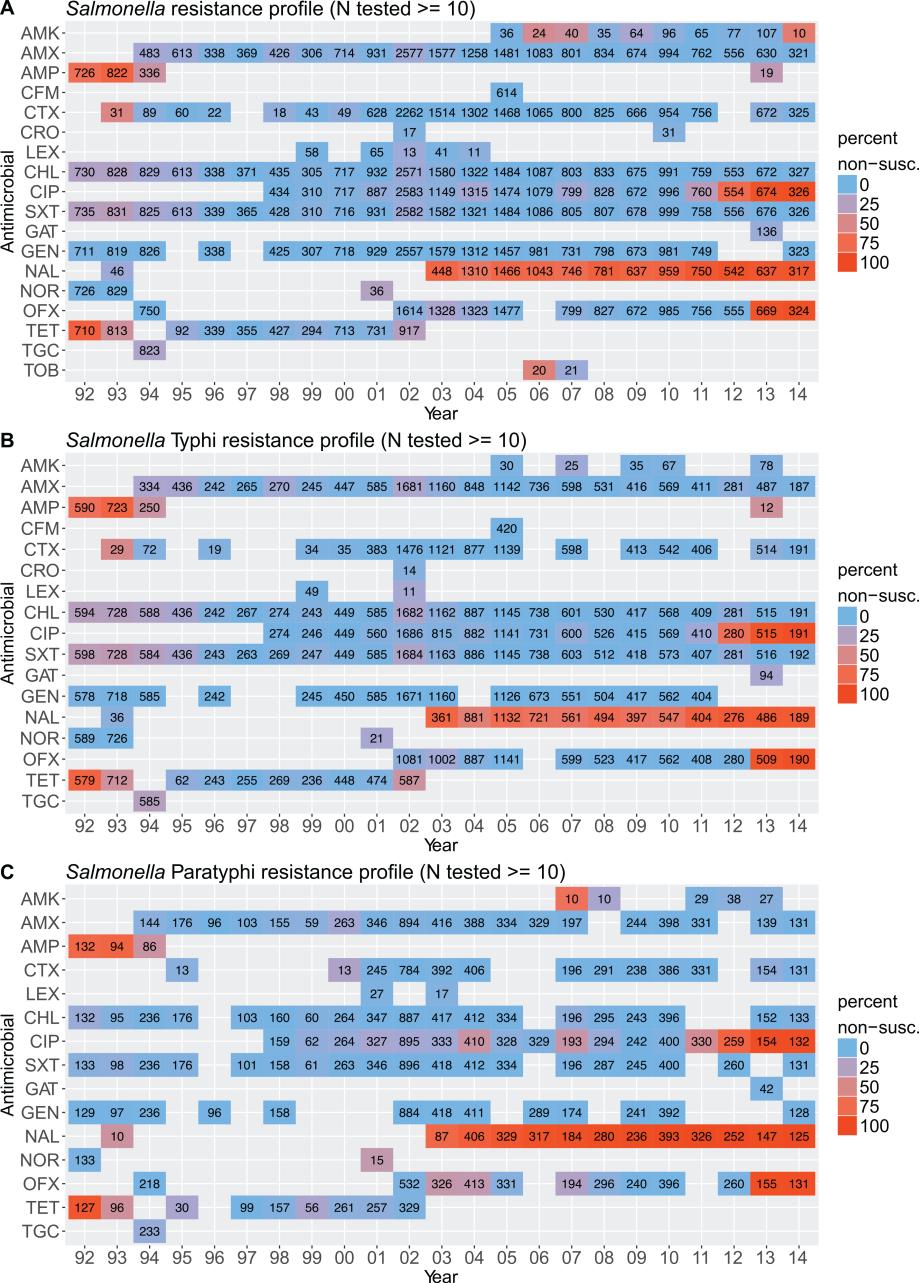
The antimicrobial susceptibility profiles of isolated *Salmonella* from 1992 to 2014. Plots summarising antimicrobial susceptibility data for all *Salmonella* (A), *S*. Typhi only (B), and *S*. Paratyphi only (C) from 1992 to 2014. The colour range on the heat map tiles indicate the proportion of non-susceptible isolates (blue, low; red; high), the number of isolates tested is indicated on the respective tile. Tiles with fewer than 10 isolates tested for the respective antimicrobial are not shown.

Trends of the antimicrobial susceptibility profiles of the *Salmonella* isolated from blood between 1992 and 2014 are shown in Fig 4. We observed a significant change in the susceptibility patterns against various antimicrobials over the study period, including increasing non-susceptibility against fluoroquinolones and increasing susceptibility against ampicillin and tetracycline for both *S*. Typhi and *S*. Paratyphi.

**Fig 5:**
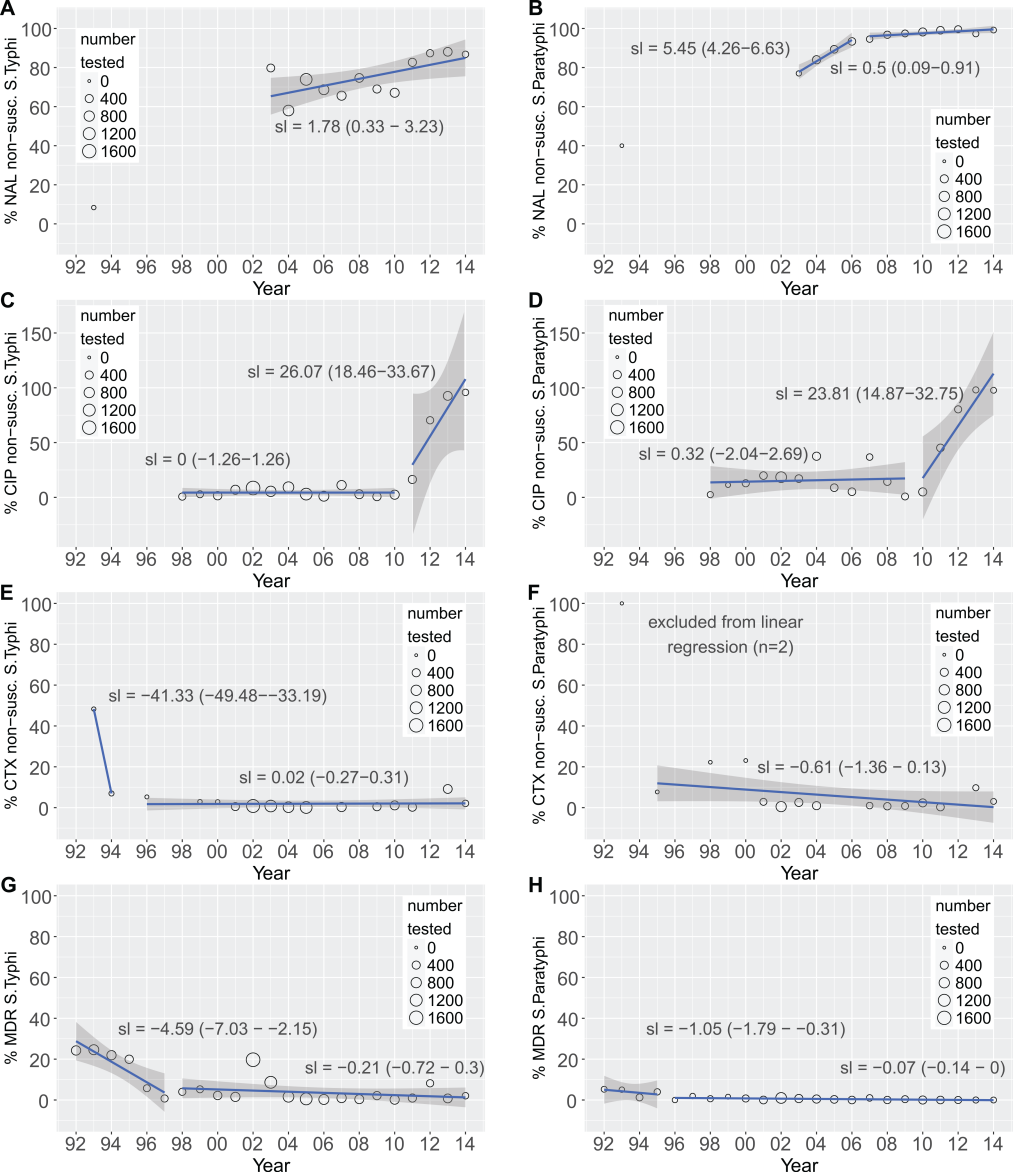
The development of non-susceptibility against selected antimicrobials and multidrug resistance. Plots showing changes in non-susceptibility against i) nalidixic acid for *S*. Typhi (A) and *S*. Paratyphi A (B), ii) against ciprofloxacin for *S*. Typhi (C) and *S*. Paratyphi (D), iii) against cefotaxime for *S*. Typhi (E) and *S*. Paratyphi A (F), and iv) multidrug resistance for *S*. Typhi (G) and *S*. Paratyphi (H) between 1992 and 2014. The linear trend-lines (blue) and 95% CI for the regression lines (shaded) are overlaid on the data points. The slope of the regression line (with 95% CI) is indicated on each plot; two regression lines are present when a breakpoint in the slope was detected.

We next investigated changes in susceptibility over the entire study period for the current first-line antimicrobials, ciprofloxacin, nalidixic acid (as a marker of reduced susceptibility against fluoroquinolones), and cefotaxime. Segmented linear regression was exploited to detect changes in regression slopes between the proportion of non-susceptible organisms and time (Fig 5). A change in slope (or “breakpoint”) was interpreted as a shift in the rate of resistance acquisition at a particular time point. Non-susceptibility against nalidixic acid was consistently high for *S*. Typhi and *S*. Paratyphi from 2002 to 2014, although a change in slope was observed for ciprofloxacin non-susceptibility between 2010 and 2011 for *S*. Typhi, and between 2009 and 2010 for *S*. Paratyphi. These breakpoints demarcated period when ciprofloxacin non-susceptibility remained low and constant and a sustained increase in ciprofloxacin non-susceptibility. For cefotaxime, a decrease in the proportion of non-susceptible *S*. Typhi was observed from 1993 to 1994, this was followed by a period of low and constant non-susceptibility from 1996 to 2014. For *S*. Paratyphi, non-susceptibility to cefotaxime was low and remained constant from 1995 to 2014

We lastly examined the proportion of MDR organisms through time (Fig 5). We observed a significant decrease in the proportion of MDR *S*. Typhi and *S*. Paratyphi from 1992 to 1997 and from 1992 to 1995, respectively. This period was followed by a period of low MDR prevalence, which was maintained throughout the study period. Notably, in 2002, a year with an atypically high number of *Salmonella* isolations, the proportion of MDR *S*. Typhi was irregularly high (19.7%), while the proportion of MDR *S*. Paratyphi isolates remained consistently low (1.0%).

## Discussion

Our study describes changes in the epidemiology and antimicrobial susceptibility patterns of *Salmonella* isolated in a Kathmandu healthcare facility from 1992 to 2014. We document (i) a significant decline in the number of *Salmonella*-positive cultures from 2002 (a year of heavy rainfall and flooding in the Kathmandu Valley) to 2014, (ii) an increase in the proportion of *S*. Paratyphi in all *Salmonella*-positive cultures between 1992 and 2014, (iii) a decline in MDR *S*. Typhi and *S*. Paratyphi, and (iv) the recent increase in fluoroquinolone non-susceptibility in both *S*. Typhi and *S*. Paratyphi.

In this comprehensive retrospective analysis of nearly 20,000 *Salmonella*-positive blood cultures we report that overall prevalence of *Salmonella* isolation from blood cultures was 8.8%. These data are consistent with data (7.2% and 13.4%) recently reported in other studies from Nepal (10,11). Over the 23-year study period, *S*. Typhi and *S*. Paratyphi A represented almost all *Salmonella*-positive cultures. However, their proportions varied over time: the proportion of *S*. Typhi ranged from >80% in 1992 to <60% in 2014. These data are consistent with studies citing a prevalence of 65.1% for *S*. Typhi and 34.9% for *S*. Paratyphi A in Alka Hospital, a neighbouring Kathmandu hospital [10]. A prevalence of 57.8% for *S*. Typhi and 42.3% for *S*. Paratyphi A was reported in a tertiary hospital in Kathmandu in 2012–2013 [11]. From 1992 to 2014, we observed a decreasing trend in the proportion of *S*. Typhi in all *Salmonella*-positive cultures; this decrease was concurrent with an increasing in the proportion of *S*. Paratyphi isolated. Similar increase have been reported in China [12] and Cambodia [13] and has been suggested to be a major shift in the aetiology of enteric fever in Asia [8,14]. The precise mechanism for this potential serovar replacement is unknown, but may be dependent on various environmental, ecological, or epidemiological factors [14]. Additionally, *S*. Paratyphi A does not harbour the Vi- polysaccharide capsule antigen, which is the key antigen used in all available *S*. Typhi vaccines in Nepal. We have only limited data on the uptake of Vi vaccine in this location, but a large Vi vaccine study was conducted in 2012 in all school-age children[15]. This program likely had a major impact on *S*. Typhi incidence and the apparent proportional increase in *S*. Paratyphi A cases. However, the vaccination program cannot entirely explain the proportional increase of *S*. Paratyphi A, which seems to have started before 2012.

The study period was not characterized by a uniform decline in *Salmonella*, but rather by a major peak in *Salmonella*-positive cultures in 2002. From 2002 to 2014, the absolute number of both *S*. Typhi and *S*. Paratyphi positive cultures declined. It is unclear whether the observed decline in *S*. *enterica* isolation from 2002 to 2014 was a return to baseline after a year of exceptionally high incidence in 2002, or if it reflected a true decrease in *Salmonella* incidence. High numbers of *S*. *enterica*-positive blood cultures were also reported from nearby hospitals in 2002. From May to July 2002, a large single-point source outbreak of MDR *S*. Typhi associated with the water supply system was reported in Bharatpur, a city ~100 km southwest of Kathmandu, with almost 6,000 confirmed or suspected cases [16]. This spike in *Salmonella* infections is likely associated with exceptional rainfall in Nepal that year, resulting in severe flooding in the Kathmandu Valley in July. We have previously shown an association between rainfall and an ability to detect *S*. Typhi and *S*. Paratyphi A DNA in the local water supplies [17]. The major *Salmonella* outbreak in the middle of the study period is a reminder that the Kathmandu Valley retains an epidemic potential for enteric fever. While we describe a decrease in Salmonella incidence since 2002, the residual enteric fever burden may flare up and cause large outbreaks under conducive conditions.

Our data additionally documented a decrease in the percentage of MDR *Salmonella* isolates; this was observed for both *S*. Typhi (1992 to 1997) and *S*. Paratyphi (1992 to 1995). Other researchers have suggested a decline in MDR-*Salmonella* in Nepal by showing that the majority of *Salmonella* isolated between 2011 and 2013 were susceptible to chloramphenicol, ampicillin, and co-trimoxazole [10,11]. Our longitudinal study confirms and expands these findings by presenting detailed kinetics in MDR *Salmonella* trends over the last 23 years: a decrease in the 1990’s followed by a long period of exceptionally low MDR prevalence.

Nalidixic acid resistance was found to be high for both *S*. Typhi and *S*. Paratyphi from 2003, the same year when antimicrobial susceptibility testing began to be performed routinely. Resistance to nalidixic acid is associated with a decline in fluoroquinolone susceptibility and the imminent emergence of fluoroquinolone resistance[18]. Indeed, we report a dramatic increase in ciprofloxacin non-susceptibility in both *S*. Typhi and *S*. Paratyphi from 2009, approaching 100% non-susceptibility by 2014. A gradual increase in ciprofloxacin non-susceptibility has been implied by various studies reporting ciprofloxacin-resistance (or non-susceptibility) for *Salmonella* in Nepal over the relevant years, ranging from 0% (*S*. Typhi) and 0.4% (*S*. Paratyphi A) non-susceptibility in 1993–1998[5], 5% (*S*. Typhi) and 13% (*S*. Paratyphi A) non-susceptibility in 1999–2003 [5], 2.8% (*S*. Typhi) and 10% (*S*. Paratyphi A) intermediate resistance in 2006–2007 [19], 8.4% resistance in 2010 [20], 0% (*S*. Typhi) and 3.3% (*S*. Paratyphi A) resistance in 2011–2012 [10], and 83.1% non-susceptibility (resistant or intermediate) in 2012–2013 [11]. Furthermore, an increase in ciprofloxacin minimal inhibitory concentrations (MIC) was reported for *S*. Typhi between 2005 and 2014, with a sharp increase in 2009 [21]. Therefore, our data confirms this trend of increasing ciprofloxacin non-susceptibility in *S*. Typhi and *S*. Paratyphi A, and details the kinetics of this transition over time. Notably, resistance to the third generation cephalosporins was low throughout the whole study period for both *S*. Typhi and *S*. Paratyphi A. Ceftriaxone remains the empirical antimicrobial of choice in this setting, which we have shown to be efficacious in those with culture confirmed enteric fever [22].

Though azithromycin is increasingly being used for the treatment of uncomplicated enteric fever, the drug was not used within the Patan Hospital treatment regimens until 2006. Within Patan Hospital it was rarely used for the treatment of enteric fever until 2014, when it was used as a “rescue drug” when the other antimicrobials failed. Due to the unavailability of break points for azithromycin against *Salmonella*, the microbiology laboratory did not perform any susceptibility tests until 2015. We are therefore unable to make any conclusions on how azithromycin resistance has evolved for *Salmonella* within our population. However, as of 2015, after the introduction of breakpoints for azithromycin on Salmonella by the CLSI, the hospital performs susceptibility testing and an ongoing surveillance monitors the MIC values.

The fluctuations in AMR profiles described in this study largely reflect changes in clinical practices. MDR decreased as empirical treatment shifted from the fist-line antimicrobials ampicillin, chloramphenicol, or co-trimoxazole to ciprofloxacin in the 1990s. As the MDR phenotype is generally acquired via IncH1 resistance plasmids in *S*. Typhi [23,24], the reduction of selective pressure after cessation of chloramphenicol, ampicillin and/or co-trimoxazole seems to induce the loss of these resistance plasmids and a reversion to a non-MDR phenotype. Subsequently, the routine use of ciprofloxacin to treat febrile diseases of presumed bacterial origin was likely associated with the dramatic increase in ciprofloxacin-non-susceptibility observed since 2009–2010. Recently, co-trimoxazole has been successfully used to treat a fluoroquinolone-resistant *S*. Typhi infection in Nepal, opening the debate about the re-deployment of first-line antimicrobials [25]. Whilst currently efficacious against fluoroquinolone resistant organisms, including the H58 variant, there is an obvious risk of re-emergence of resistance with the reintroduction of the older antimicrobials, therefore methods such as antimicrobial cycling could be considered [21].

Our findings from almost 20,000 *Salmonella enterica* isolates highlight the need to review the current treatment protocols of enteric fever from fluoroquinolones as recommended by the WHO and local health ministries. There is sufficient evidence to use azithromycin or cephalosporins as the first antimicrobial therapy instead of fluoroquinolones [22,26]. With the re-emergence of efficacy in older antimicrobials used to treat enteric fever, they could be considered for use, but perhaps in combination with other antimicrobials to avoid an early re-emergence of resistance to these antimicrobials. However, treatment guidelines should be meticulously guided by microbiology data.

Our analysis though comprehensive has a few limitations, being a passive surveillance focused on one particular hospital. One of our primary limitations is the use of different susceptibility guidelines used for resistance interpretation over time as the CLSI guidelines were unavailable locally prior to 2003. We were not able to provide a consistent interpretation through the retrospective use of CLSI guidelines as the isolates were not stored and the disc diameters were not recorded. Another limitation is the population that is seen in the hospital, though the catchment area of the hospital has remained the same throughout the study period, many other health care facilities have opened up over time within this area where a certain subset population would go to seek medical care. However, Patan Hospital, being the biggest general hospital in the area still does see a majority of the patients within the catchment area. Hospital records showing individuals seeking care in Patan Hospital has increased consistently over the years, in accordance to the increase of the local population.

## Supporting information

S1 Fig The annual distribution of blood culture results. Annual counts (A) and percentages (B) of *Salmonella*-positive cultures, cultures positive for other bacteria, negative cultures and contamination in all blood cultures taken between 1992 and 2014.
(EPS)

S2 Fig The annual distribution of *Salmonella*-positive cultures. Percentage of *Salmonella*-positive cultures among all positive cultures from 1992 to 2014. Symbol size is proportional to the number of positive cultures. A linear regression trend-line (blue) and confidence interval (shaded) are overlaid from 2002 to 2014 to illustrate the decline in the proportion of *Salmonella* in all positive cultures. The slope (and 95% CI) of the regression line is indicated on the graph.
(EPS)

S3 Fig Autocorrelation function of Salmonella cases. Autocorrelation function (ACF) for monthly time series for *Salmonella* (Tyhi, Paratyphi and spp) (A), *S*. Typhi (B) and *S*. Paratyphi (C). One lag represents one year divided in 12 months.
(EPS)

S1 Data Microbiology laboratory report on *Salmonella* spp from April 1992 to December 2014 from Patan Hospital. (CSV)
